# Rationally Designed Magnetic Nanoparticles for Cochlear Drug Delivery: Synthesis, Characterization, and In Vitro Biocompatibility in a Murine Model

**DOI:** 10.1097/ONO.0000000000000013

**Published:** 2022-07-20

**Authors:** Mukund M. Goyal, Nancy J. Zhou, Philippe F. Y. Vincent, Elina S. Hoffman, Shiv Goel, Chao Wang, Daniel Q. Sun

**Affiliations:** 1 Department of Chemical and Biomolecular Engineering, Johns Hopkins University, Baltimore, MD; 2 School of Medicine, Johns Hopkins University, Baltimore, MD; 3 Department of Otolaryngology – Head and Neck Surgery, Johns Hopkins University, Baltimore, MD.

**Keywords:** Biocompatibility, Drug delivery, Magnetic nanoparticles, Organ of Corti

## Abstract

**Hypothesis:**

Magnetic nanoparticles (MNPs) for cochlear drug delivery can be precisely engineered for biocompatibility in the cochlea.

**Background:**

MNPs are promising drug delivery vehicles that can enhance the penetration of both small and macromolecular therapeutics into the cochlea. However, concerns exist regarding the application of oxidative, metal-based nanomaterials to delicate sensory tissues of the inner ear. Translational development of MNPs for cochlear drug deliver requires specifically tuned nanoparticles that are not cytotoxic to inner ear tissues. We describe the synthesis and characterization of precisely tuned MNP vehicles, and their in vitro biocompatibility in murine organ of Corti organotypic cultures.

**Methods:**

MNPs were synthesized via 2-phase ligand transfer process with precise control of nanoparticle size. Core and hydrodynamic sizes of nanoparticles were characterized using electron microscopy and dynamic light scattering, respectively. In vitro biocompatibility was assayed via mouse organ of Corti organotypic cultures with and without an external magnetic field gradient. Imaging was performed using immunohistochemical labeling and confocal microscopy. Outer hair cell, inner hair cell, and spiral ganglion neurites were individually quantified.

**Results:**

Monocore PEG-MNPs of 45 and 148 nm (mean hydrodynamic diameter) were synthesized. Organ of Corti cultures demonstrated preserved outer hair cell, inner hair cell, and neurite counts across 2 MNP sizes and doses, and irrespective of external magnetic field gradient.

**Conclusion:**

MNPs can be custom-synthesized with precise coating, size, and charge properties specific for cochlear drug delivery while also demonstrating biocompatibility in vitro.

A primary challenge in the treatment of inner ear conditions such as sensorineural hearing loss is the safe and effective delivery of therapeutics directly into the inner ear. Although many candidate pharmaceutics, ranging from small molecules to macromolecular biologics, have demonstrated promising pharmacodynamic potential (1–7), anatomical barriers such as the round window membrane pose critical diffusion barriers to effective drug penetration into the cochlea (8). Many delivery systems have been investigated to enhance the local delivery of therapeutics into the cochlea, such as micro-osmotic pumps (9,10), drug-eluting cochlear implant electrodes (11–13), microneedles (14,15), hydrogels (16), and nanoparticles (17,18), among others. In particular, magnetic nanoparticles (MNPs) have unique chemico-physical properties that render them highly attractive as a minimally invasive and re-doseable platform for cochlear drug delivery (19,20). Comprised of iron oxide nanocores surrounded by a polymeric coating, they are able to undergo magnetically assisted transport across biological tissues while releasing therapeutic payloads. Recently, MNPs loaded with adenoviral vectors carrying a BDNF-encoding gene has been shown to penetrate into the cochlea after intratympanic injection and result in BDNF gene expression in the cochlea with attenuation of noise-induced hearing threshold shifts in rats (21). Similarly, other studies have investigated the potential of MNPs as drug delivery vehicles for small molecules, nucleic acids, and proteins into the cochlea (22,23).

In the engineering of MNPs for cochlear drug delivery, particle coating has important implications for drug elution, nanoparticle transport, and biocompatibility (24). Due to hydrophobicity of Fe_3_O_4_ nanocores and potential for oxidative injury as MNPs undergo metabolism by cells, the coating of MNPs require special design considerations to achieve both biocompatibility and optimal tissue transport (25). Thick coating can improve drug payload capacity and controlled release, but may result in increased drag in magnetically assisted transport, while thin coating may increase tissue toxicity due to exposure of iron oxide nanocores (26). While coating type (24), surface charge (27), and functionalization (28) have been studied previously, there is significant heterogeneity in the particle characteristics used, which can have important biological implications in the ear. For instance, studies (29) have shown that endocytosis mechanisms are highly dependent on particle size and, therefore, broad size distributions in particle batches can confound experimental results. Furthermore, while many MNP studies (21,23,30) have demonstrated a therapeutic benefit when using drug payloads, few studies have specifically examined the biocompatibility of the MNP vehicle in the inner ear (31). Nanotoxicity is an active area of investigation across multiple organ systems including the central nervous system (CNS) (25); however, the extent to which delicate sensory structures of the cochlea may be susceptible to potential oxidative injury from iron oxide nanocores has not been specifically studied.

In this study, we present an MNP synthesis strategy that allows coating thickness to be precisely controlled, by grafting differential PEG chain lengths to iron oxide nanocores (Fig. 1A, B). We characterize the physicochemical properties of synthesized MNPs and assess their differential in vitro biocompatibility in an organotypic culture by both MNP dose and presence of a magnetic field gradient, with findings relevant for cochlear applications.

**FIG. 1.**
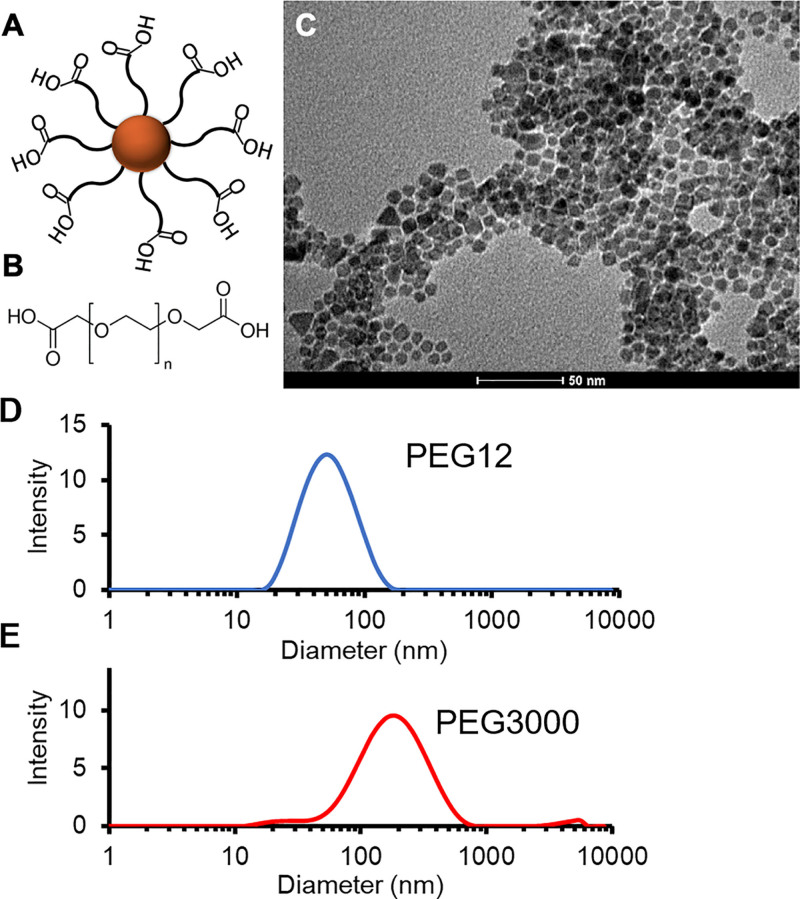
*A*, Core-shell MNPs comprised of an Fe_3_O_4_ monocore with brush-type PEG coating. *B*, Hydrodynamic size can be tuned via length of PEG chain grafted to nanocore. *C*, Transmission electron microscopy demonstrates highly homogeneous and monodispersed Fe_3_O_4_ monocores of approximately 7 nm each. *D and E*, PEG12 and PEG3000 MNPs are found to have mean hydrodynamic diameters of 45 (Polydispersity index, PDI 0.21) and 148 nm (PDI 0.28), respectively, via dynamic light scattering.

## METHODS

### Synthesis of Iron Oxide Nanoparticles

Iron oxide nanoparticles of size ~7 nm were synthesized by thermal decomposition of Iron (III) acetylacetonate (Fe(acac)_3_) using organic phase synthesis as described previously (32). For synthesis of ~7 nm Fe_3_O_4_ nanoparticles, 2 mmol (0.763 g) of Fe(acac)_3_ is mixed with 10 mL of benzyl ether and 10 mL of oleylamine. The mixture was heated for 1 hour at 110°C for dehydration followed by reflux at 300°C for 30 minutes. The precipitate was then collected, and nanoparticles were washed with ethanol 3 times and then re-dispersed in hexanes. The nanoparticles were then stabilized by adding ~0.25 mL of oleic acid.

### Surface Modification

The oleylamine on the surface of synthesized nanoparticles was replaced with a poly ethylene glycol diacid-12 molecule chain (PEG12, Sigma Aldrich) to make NP-PEG12 and *α, ω*-Bis{2-[(3-carboxy-1-oxopropyl) amino] ethyl} polyethylene glycol-relative molecular weight 3000 (PEG-3000, Sigma Aldrich) to make NP-PEG3000 and disperse them in water using procedure as described previously (32). PEG12 (Mr = 690) and PEG3000 (Mr = 3000) are comprised of linear chain of ~12 and ~61 ethylene glycol monomers, respectively, and are differentially grafted to Fe3O4 monocores to generate thin and thick coatings, respectively. In a typical experiment, 4.9 mg of O, O′-bis(2-carboxymethyl) dodecaethyl glycol (to make NP-PEG12) or 20 mg of *α, ω*-Bis{2-[(3-carboxy-1-oxopropyl) amino] ethyl} polyethylene glycol (to make NP-PEG 3000), 2 mg N-hydroxysucccinimide (NHS), 3 mg 1-ethyl-3-(3-dimethylaminopropyl) carbodiimide (EDC), 1.27 mg dopamine hydrochloride, 10 mg Na_2_CO_3_, 1 mL CHCl_3_, and 1 mL DMF were mixed under argon protection for 3 hours. 5 mg of Fe_3_O_4_ particles dissolved in 1 ml of chloroform were then added and allowed to stir for overnight (~12–14 hours) under argon protection. The particles were then collected using hexanes and separated using centrifugation at 10,000 rpm for 5 minutes. The supernatant solution was then removed, and particles were dried in antechamber for 1 minute to remove any organic solution in the tube. The nanoparticles were then dispersed in 5 mL of phosphate buffered saline (PBS) water followed by sonication for 20–30 minutes to allow complete dispersion in water. The extra amount of chemicals present in the solution were removed by dialysis. To perform dialysis, the nanoparticle solution was injected into 12 mL Slide-A-Lyzer Dialysis Cassette with a 10K MWCO rating (Thermo Fisher Scientific) and stirred on magnetic stirrer at 500 rpm for 24 hours in DI water.

### Characterization and Imaging

Transmission electron microscopy (TEM) images were acquired on a 120 kV, FEI Tecnai 12 Twin Microscope at 100 kV Voltage. TEM images were acquired for Fe_3_O_4_ NPs in Hexanes, and Fe_3_O_4_-PEG12, Fe3O4-PEG3000 NPs in PBS. Dynamic light scattering and zeta potential measurements were made using Zetasizer Nano S- Malvern Panalytical to find approximate size of Nanoparticles and their change in size and overall charge upon pegylation with PEG12 and PEG3000 chains.

### Organ of Corti Culture

Animal protocols were approved by institutional Animal Care and Use Committee. The mid-turn of each organ of Corti was microdissected from neonatal (P3–P5) C57BL/6J mouse pups and individually cultured in growth media supplemented with serum, inoculated with NT3 and BDNF, and coincubated with PEG12 MNPs (0.1 or 0.5 mg Fe/mL), PEG3000 MNPs (0.1 or 0.5 mg Fe/mL), or neomycin/kainic acid (0.1 mM/5 mM) for 72 hours (n = 3 for each condition), and with or without an external magnetic field gradient (neodymium permanent magnet, 0.3 T at surface) oriented orthogonal to the culture dish. As bare Fe_3_O_4_ nanoparticles are not water soluble, neomycin/kainic acid were used to ablate HCs and neurites.

### Immunohistochemistry and Quantification

After 72 hours, cultures were fixed and labeled with antineurofilament (NF200, Sigma Aldrich) and anti-Myosin VI (abchem) antibodies for neurite and hair cell labeling, respectively. Images of the cultures were acquired via confocal microscopy (Zeiss LSM 700) at 10× objective. inner hair cell (IHC), outer hair cell (OHC), and spiral ganglion neuron (SGN) neurite counts were individually quantified using ImageJ. Cell counts were normalized by distance along the organ of Corti to 0.1 mm. Statistical testing for between-group differences was performed using ANOVA and Tukey’s test (Stata 17, College Station, TX).

## RESULTS

Custom-synthesized, monocore MNPs were found to be monodispersed in water with mean core size of 7nm, on TEM imaging (Fig. 1C). Grafting with differential PEG chain lengths resulted in mean hydrodynamic diameters of 45nm (polydispersion index, PDI, 0.21) and 148nm (PDI, 0.28) for PEG12 and PEG3000-coated MNPs, respectively (Fig. 1D). Zeta potentials were measured to be −38.5 and −29.7mV for PEG12 and PEG3000-coated nanoparticles, respectively.

**FIG. 2.**
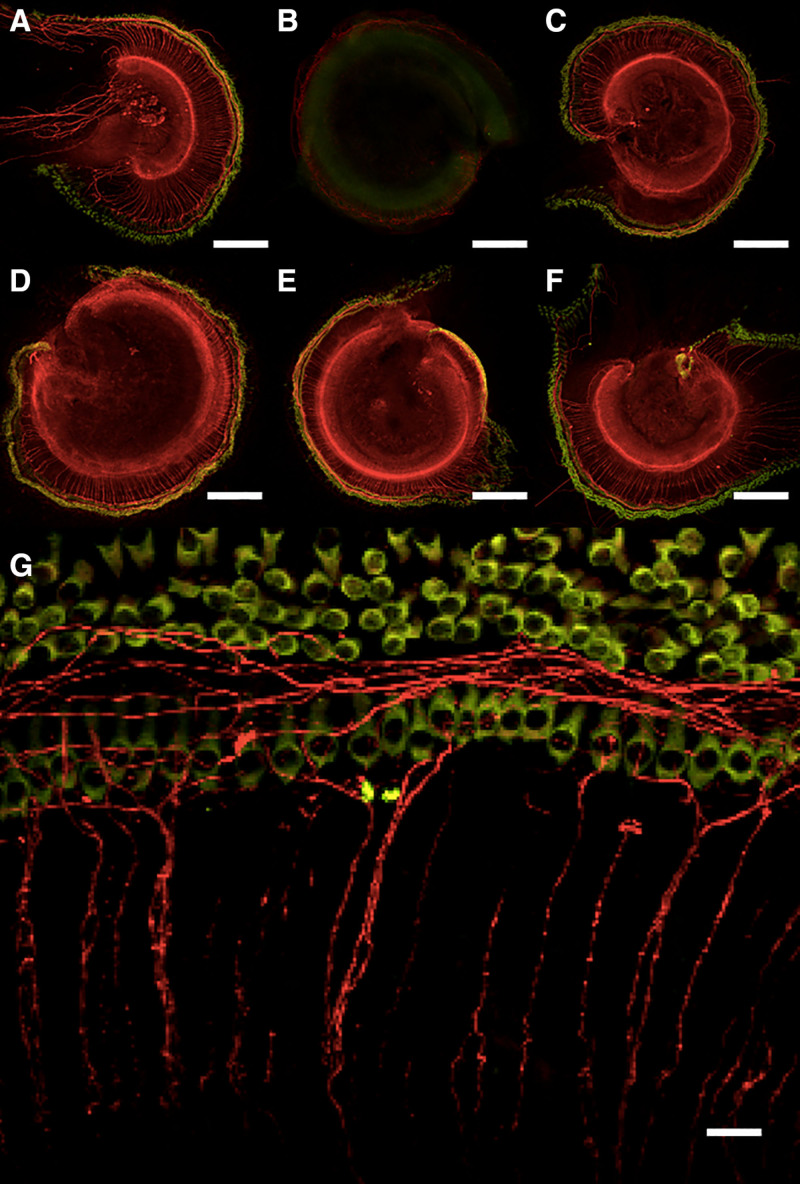
Confocal microscopy with staining for spiral ganglion neurons (red) and hair cells (green). *A*, Saline. *B*, Neomycin/kainic acid. *C*, PEG3000 0.5 mg Fe/mL. *D*, PEG3000 0.1 mg Fe/mL. *E*, PEG12 0.5 mg Fe/mL. *F*, PEG12 0.1 mg Fe/mL. *G*, Saline control magnified for quantification of individual cells. Scale bar: 200 µm in (A–F), 20 µm in (G).

Figure 2 shows representative organ of Corti cultures under various experimental conditions imaged using confocal microscopy. Anti-NF200 and anti-Myosin VI antibodies demonstrate robust and specific staining for neurites and hair cells (both inner and outer), respectively. Histologically, SGN neurites, IHCs, and OHCs remained intact after 72-hour incubation with both PEG12- and PEG3000-coated MNPs at 0.1 and 0.5 mg Fe/mL doses. In contrast, incubation with neomycin and kainic acid led to severe disruption of both hair cells and SNG neurites.

**FIG. 3.**
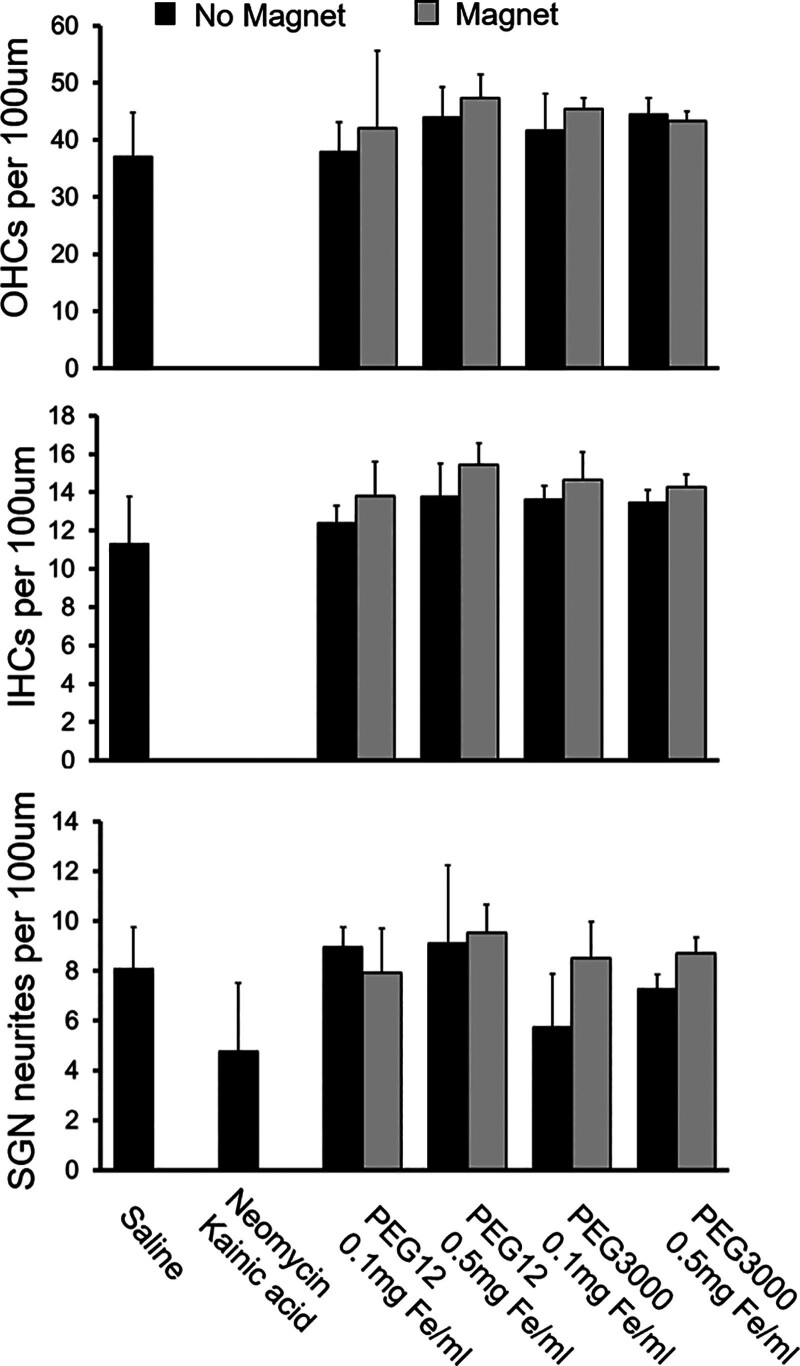
Cellular quantification of organ of Corti cultures (n = 3 per condition) demonstrate lack of significant differences in OHC, IHC, and SGN neurite counts across differential MNP size and dose, as well as in the presence of an external magnetic field gradient. In contrast, neomycin/kainic acid demonstrated complete ablation of IHCs and OHCs, and partial ablation of SGN neurites. As bare Fe_3_O_4_ nanocores are not water soluble, the cytotoxicity of uncoated metal oxide nanocores could not be specifically quantified.

OHC, IHC, and SNG neurites were specifically quantified to evaluate the effects of coating thickness and MNP dose in organotypic cultures (Fig. 3). Relative to saline, neomycin and kainic acid led to complete ablation of IHCs and OHCs, and partial ablation of SGN neurites. In contrast, OHC, IHC, and SGN neurite counts were preserved (*P* > 0.05) when cultures were coincubated with PEG12- or PEG3000-coated nanoparticles at both investigated doses. Furthermore, we quantified OHC, IHC, and SGN neurites when an external magnetic field gradient was applied to MNP cultures. Visual inspection of organ of Corti cultures demonstrated magnetically mediated concentration of MNPs on the tissue surface, in contrast to cultures without a magnetic field gradient. However, OHC, IHC, and SGN neurite quantification demonstrated preserved cell counts similar to saline (*P* > 0.05).

## DISCUSSION

We describe an MNP synthesis process that allows both particle size and coating thickness to be precisely tuned for biological applications and demonstrate the short-term, in vitro biocompatibility of custom-synthesized MNPs in a mouse cochlea organotypic culture. Although iron oxide nanoparticles have been FDA-approved for systemic applications related to the treatment of anemia and as MR-contrast agents (33,34), their specific tissue toxicity in drug delivery applications have not been well-studied. Cytotoxicity related to metal-based nanoparticles, including iron oxide MNPs, are primarily mediated through the oxidative effects of the metal nanocores leading to generation of reactive oxygen species, which can lead to apoptosis (25). Studies also suggest that, because cellular uptake of MNPs primarily occurs via endocytosis and that they are then localized to lysosomes, lysosomal dysfunction due to MNP-induced reactive oxygen species generation is the primary mechanism that leads to activation of autophagy pathways (35). In emerging biomedical applications of MNPs, nanoneurotoxicity in the CNS is an area of active investigation, but the specific cellular and physiologic impact of MNP uptake and reactive oxygen species generation in CNS cells remain poorly understood (25,36). The connection of the cochlea to the CNS via both the cochlear nerve and the cochlear aqueduct, lack of a robust system of circulating macrophages or microglia that clear nanoparticles in systemic circulation or the CNS (37), respectively, and combined with the delicate sensory structures highly vulnerable to oxidative injury, render the inner ear a unique microenvironment for the application of MNPs and where tissue biocompatibility requires specific study.

We found that custom-synthesized MNPs used in this study did not demonstrate organ of Corti cytotoxicity across 2 regimes of hydrodynamic size (Fig. 3). The hydrodynamic radii used in this study align with the low and high ranges in the size of core-shell nanoparticles used in other studies in the inner ear (22,23,38–40). Although studies have suggested that particle size is an important mediator of cytotoxicity (25,41), it has been difficult to deconvolute the differential impact of nanoparticle core and hydrodynamic sizes, as well as surface charge, on cellular toxicity (42). By precisely tuning coating thickness, the custom-synthesized nanoparticles used in this study have identical core diameter and similar zeta potential, while differing in hydrodynamic size (Fig. 1). Coating thickness is an important consideration in the design of MNPs for drug delivery applications due to their unique physicochemical properties. In contrast to inert nanoparticles, differential core and hydrodynamic radii have differential impact on MNP transport, as the magnetic force exerted on an MNP is a function of its core size while fluid drag and tissue interactions are functions of hydrodynamic size (26,43). Furthermore, coating thickness also has important implications for payload capacity and drug elution rate when designing MNPs as drug delivery vehicles (24). Therefore, the extent to which coating thickness and vis-a-vis, hydrodynamic size, may impact biocompatibility in the inner ear is critical to characterize.

In designing MNPs used in this study, a brush-type coating was synthesized, which is typically denser and sturdier than matrix-type coatings as one end of each polymer chain is covalently grafted to the nanocore (24). Studies (24,25,44) have suggested that brush-type coatings may be more effective at stabilizing Van der Waals interactions between nanocores to decrease agglomeration and also may impact intracellular degradation of the coating and consequent exposure of the iron oxide nanocore to affect biocompatibility. While studies have also demonstrated dose-related cytotoxicity in vitro and in vivo (42), specific dosing for MNPs as drug delivery vehicles in inner ear applications remains to be determined and will likely depend on both the specific vehicle and the specific drug payload. Dosing in inner ear-related MNP studies have been heterogeneous and span a broad range, and the doses examined in this study are also within that range (21–23,38,42,45). In addition, the effective concentration of MNPs at the cell surface is also expected to be even higher in culture conditions with an external magnetic field gradient.

While no cytotoxicity was observed in short-term organotypic cultures, future studies will focus on specific MNP-cellular interactions using fluorescently labeled MNPs and assessment of molecular biomarkers related to oxidative stress and protein damage, for instance. Further in vivo toxicity and biocompatibility studies, including assessment of auditory physiology and comparison of nanoparticles with differential surface coating and charge, will also be required. These studies will also allow examination of the basal and apical regions of the cochlea and across age ranges, which may show differential vulnerability to MNP-mediated ototoxicity, and the ability of these custom-engineered MNPs to traverse the round window membrane. Although the lack of operator blinding may lead to bias in cellular quantification, reassuringly, hair cell counts obtained from in vitro cultures in the current study concord with those from previous in vivo studies focused on therapeutic delivery (23). Finally, as bare iron oxide nanoparticles are not water soluble, their specific cytotoxicity and the extent to which it is mitigated by differential PEG coating could not be specifically studied.

In summary, custom-synthesized MNPs with precisely tuned physicochemical properties represent a promising approach for cochlear drug delivery. We report the synthesis and characterization of core-shell MNPs with differential hydrodynamic size via tuning of PEG coating thickness. In vitro biocompatibility studies using murine organ of Corti organotypic cultures suggest lack of cytotoxicity to OHCs, IHCs, and SGN neurites across a range of nanoparticle size and dosing.

## ACKNOWLEDGMENT

The authors thank Elisabeth Glowatzki, PhD, and Charles C. Della Santina, PhD, MD, for their assistance with this work. The authors also gratefully acknowledge Elisabeth Glowatzki for assistance with tissue culture, and the Capita Foundation, Rubenstein Hearing Research Fund, and Johns Hopkins University Discovery Award for their generous support.

## FUNDING SOURCES

None declared.

## CONFLICT OF INTEREST

None declared.

## DATA AVAILABILITY STATEMENT

The datasets generated during and/or analyzed during the current study are not publicly available, but are available from the corresponding author on reasonable request.
